# Efficacy of empirical Ciprofloxacin or Cefixime plus Metronidazole therapy for the treatment of liver abscess: a randomized control clinical trial

**DOI:** 10.1038/s41598-024-59607-1

**Published:** 2024-05-20

**Authors:** Lakshmi Priya G, Deba Prasad Dhibar, Atul Saroch, Navneet Sharma, Vishal Sharma, Nipun Verma, Sreedhara Bettadahally Chaluvashetty, Ajay Prakash, Harsimran Kaur

**Affiliations:** 1 Department of Internal Medicine, Nehru Hospital PGIMER, F-Block, Chandigarh, 160012 India; 2 Department of Gastroenterology, PGIMER, Chandigarh, India; 3 Department of Hepatology, PGIMER, Chandigarh, India; 4 Department of Radio Diagnosis and Imaging, PGIMER, Chandigarh, India; 5 Department of Pharmacology, PGIMER, Chandigarh, India; 6 Department of Microbiology, PGIMER, Chandigarh, India

**Keywords:** Liver abscess, Ciprofloxacin, Cefixime, Empirical oral antimicrobial, Gastrointestinal diseases, Hepatology, Medical research, Antimicrobials

## Abstract

Liver abscess is a potentially life-threatening medical emergency. Prompt empirical antimicrobial with or without percutaneous aspiration or drainage is therapeutic. The rational for using empirical intravenous broad-spectrum antimicrobials upfront instead of oral Fluoroquinolone or Cephalosporin is contentious. In this double blind randomized control clinical trial 69 participants received Ciprofloxacin (500 mg q 12 hourly) and 71 participants received Cefixime (200 mg q 12 hourly) orally for 2 weeks. Both the group received oral Metronidazole (800 mg q 8 hourly) for 2 weeks and percutaneous drainage or aspiration of the abscess was done as per indication and followed-up for 8 weeks. Out of 140 participants, 89.3% (N = 125) achieved clinical cure, 59 (85.5%) in Ciprofloxacin group and 66 (93%) in Cefixime group (p = 0.154). Mean duration of antimicrobial therapy was 16.2 ± 4.3 days, 15.1 ± 4.5 days in Ciprofloxacin group and 16.0 ± 4.2 days in Cefixime group (p = 0.223). Total 15 (10.7%) participants had treatment failure, 10 (14.5%) in Ciprofloxacin group and 5 (7.0%) in Cefixime group (p = 0.154). The most common reason for treatment failure was need of prolong (> 4 weeks) antimicrobial therapy due to persistent hepatic collection requiring drainage, which was significantly (p = 0.036) higher in Ciprofloxacin (14.5%, N = 10) group, compared to the Cefixime (4.2%, N = 3) group. In conclusion, both, the Ciprofloxacin or Cefixime plus Metronidazole for duration of 2–3 weeks were efficacious as empirical oral antimicrobial regimen along with prompt percutaneous drainage or aspiration for the treatment of uncomplicated liver abscess with similar efficacy. Oral Cefixime was better than Ciprofloxacin in term of lesser chance of treatment failure due to persistent collection which is required to be investigated further in larger clinical trial.

**Trial registration**: clinicaltrials.gov PRS ID: NCT03969758, 31/05/2019.

## Introduction

Liver abscess is a potentially life-threatening, frequently encountered medical emergency with significant morbidity and mortality. Prompt empirical antimicrobial therapy with or without percutaneous aspiration or drainage of the abscess is therapeutic. Liver abscess is predominantly due to amoebic, bacterial or mixed polymicrobial infection of the liver which gets localized to form an abscess. An empiric antimicrobial regimen for liver abscess should cover enteric gram-negative bacilli, streptococci, anaerobes and antamoeba histolytica^1^. Presently a Fluoroquinolone (Ciprofloxacin, Levofloxacin) or a third or fourth generation Cephalosporin (Cefixime, Ceftriaxone, cefepime) or a Beta-lactam-beta-lactamase inhibitor combination (piperacillin-tazobactam or ticarcillin-clavulanate) or a Carbapenem (Imipenem-cilastatin, Meropenem, Doripenem, Ertapenem) are being used in combination with or without Metronidazole as the empirical intravenous antimicrobial regimen for the treatment liver abscess^1^. Justification for using intravenous broad-spectrum antimicrobial combinations upfront, instead of oral Ciprofloxacin or Cefixime in empirical antibiotic regimen for the treatment of liver abscess is contentious due lack of clinical trial based evidence^2,3^. There is also lack of clinical trial to evaluate the efficacy of oral antimicrobials for the treatment of liver abscess. Injudicious use of intravenous broad-spectrum antibiotics for the treatment of liver abscess with or without complication may also contribute to future rise in antibiotic resistance. There is no medical literature available, demonstrating the superiority of one combination of empirical antimicrobial over the other. In our practice we use Cephalosporins, Fluoroquinolones and Piperacillin-tazobactam which apparently have similar efficacy or outcomes. At present, there is no randomized control clinical trial (RCT) available to evaluate and compare the efficacy of the oral antimicrobial regimens for the treatment of liver abscess as well as there is no specific treatment guideline for the use of empirical antimicrobial regimen. Both ciprofloxacin and Cefixime are effective oral antibiotics, as they are well-absorbed orally with good oral bioavailability and achieve plasma concentration well above the minimal inhibitory concentrations require for the killing of the microorganism^4,5^. But the growing antimicrobial resistance to ciprofloxacin is also a concern. Using intravenous antibiotics upfront, for the treatment of liver abscess in patients who can take orally may unnecessarily increase the duration of hospital stay and the cost of therapy, as well as the risk of hospital acquired infection. The present study was to evaluate and compare the efficacy of empirical oral Ciprofloxacin or Cefixime plus Metronidazole therapy for the treatment of liver abscess.

## Method

### Aim

Aim of the study was to evaluate and compare the efficacy of empirical oral Ciprofloxacin or Cefixime plus Metronidazole therapy for the treatment of uncomplicated Liver abscess.

### Site of study

The study was conducted in emergency medical outpatient department (EMOD) of post graduate institute of medical education and research (PGIMER), a tertiary care centre in northern India under collaboration with the department of Internal Medicine, Gastroenterology, Hepatology, Radio diagnosis & Imaging, Pharmacology and Microbiology.

### Study design

In this randomized control double blind clinical trial individuals presented with newly diagnosed liver abscess were screened for enrollment and randomized into Ciprofloxacin and Cefixime groups as per inclusion and exclusion criteria, denoting the two different combination of empirical oral antibiotics therapy for the treatment of liver abscess. Both the groups were advised for oral Metronidazole and standard care along with percutaneous drainage or aspiration of the abscess as per indication and followed up for 8 week physically or telephonically as and when required. The Institutional Ethics Committee (IEC) reviewed and approved the study protocol (approval ID: INT/IEC/2019/001028) and Indian Council of Medical Research (ICMR) guideline for clinical research was followed. Written informed consent was obtained from all subjects and/or their legal guardian for the enrollment in the study. The study was registered with clinicalTrial.gov (clinicaltrials.gov PRS ID: NCT03969758, 31/05/2019).

### Study duration

The study was carried out during the period of May 2019 to July 2020.

### Inclusion and exclusion criteria

Irrespective of sex (male or female) with age ≥ 18 years, all the symptomatic patients of liver abscess confirmed with radiology imaging, either by ultrasonography (USG) or computed tomography (CT) scan was screened for enrollment in the study. Patients with past history of liver abscess, chronic kidney disease (CKD), history of hypersensitivity to either Ciprofloxacin or Metronidazole or Cefixime were excluded from the study. Patients with shock (blood pressure < 90/60 mmHg), acute respiratory distress syndrome (ARDS, PaO2/FiO2 ≤ 300), encephalopathy (altered sensorium with GCS < 15), acute kidney injury (AKI, increase in serum creatinine to ≥ 1.5 times from the baseline) and pregnancy at presentation were also excluded from the study. Patients who already received antibiotics for more than 48 h prior to the admission and who were not able to take orally were also excluded from the study. Further patients who were receiving blood thinners like anti-platelets, anti-coagulation agents within 4 weeks of presentation, were also excluded from the study due to higher risk for bleeding complication in the course of percutaneous aspiration or drainage of the liver abscess.

### Method and intervention

In this double blind clinical trial, all the patients presenting with newly diagnosed liver abscess were admitted and evaluated at EMOPD and subsequently screened and enrolled in the study as per inclusion and exclusion criteria after getting written informed consent. The participants were randomly assigned into Ciprofloxacin and Cefixime group which received oral Ciprofloxacin (500 mg q 12 hourly) or Cefixime (200 mg q 12 hourly) respectively for 2 weeks. Both the group received oral Metronidazole (800 mg q 8 hourly) for 2 weeks and percutaneous drainage or aspiration of the abscess was done as per indication.

Randomization, allocation and treatment concealment was executed by the pharmacologist through computer generated randomization. Both the participants and the principal investigator were kept unaware of treatment (Ciprofloxacin or Cefixime) allocation. The diagnosis of liver abscess was established on clinical presentations (fever, jaundice, pain abdomen, anorexia, nausea, vomiting, diarrhoea, cough, weight loss) and USG or CT scan imaging confirming abscess formation in the liver. Details of clinical history and examination were noted and baseline blood investigations (complete blood count, coagulation profile, electrolytes, renal function and liver function test, Arterial blood gas analysis) of all the participants were done for risk stratification and to devise further plan of management. Blood for bacterial culture and amoebic serology were sent prior to the initiation of empirical antibiotics therapy. USG was done in all participants for the diagnostic as well as percutaneous aspiration or drainage purpose. CT scan was performed in case of doubt regarding the diagnosis or suspected complication in USG. Data was assembled for each participant in a predesigned case record form.

For management, initial prime concern was to secure airway, breathing and circulation. To safeguard hemodynamic stability, crystalloids infusion was given as and when required. Once diagnosis of liver abscess was confirmed, the participants fulfilling the desired inclusion and exclusion criteria were randomly assigned into the two study groups after obtaining written informed consent either from the patients or legal representative. Percutaneous aspiration or drainage of the liver abscess was done when there was enough liquid content/pus which was amenable for aspiration or drainage by the expert intervention radiologist. Percutaneous drainage or aspiration was executed in liver abscess with size of ≥ 5 cm, left lobe abscess and when there was risk for impending rupture. Supportive care like antipyretics, analgesics and hydration was given as per requirement. Participants with coagulopathy received Fresh frozen plasma (FFP) transfusion and vitamin K injection to achieve coagulation-profile target of PTI > 65% or INR < 1.5. Aspirated or drained pus was processed for microscopy, culture & sensitivity in the department of microbiology of the institute. If during the course pus culture demonstrated growth of microorganism which was not sensitive to Ciprofloxacin in Ciprofloxacin-group or not sensitive to Cefixime in Cefixime-group, the antibiotic was changed as per sensitivity pattern and the case was defined as treatment failure. Pigtail catheter was removed when there was no drainage of pus for more than 48 h with USG showing no significant drainable collection in the liver. After 2 weeks of empirical antibiotic therapy, asymptomatic participants with persistent drainage of pus and USG indicating significant drainable collection in the liver, the same combination of antimicrobial therapy was continued for another 2 weeks or till the PCD was removed. Any participant with persistent drain output for more than 4 weeks was also defined as treatment failure and was shifted to intravenous antimicrobials. When participants did not exhibit any symptomatic improvement even after 72 h of empirical antibiotics and percutaneous aspiration or drainage, developed new collection or organ dysfunction the antibiotics were modified to IV Beta-lactam-beta-lactamase inhibitor combination or Carbapenem or as per culture sensitivity report of the drained pus. All the participants were followed up for 8 weeks and the outcomes of both groups was compared. Adverse drug reaction (ADR) incurred in the participants during the study was notified to ADR Monitoring Centre, PGIMER, under the Pharmacovigilance Programme of India (PvPI), National Coordination Centre (NCC)–Indian Pharmacopoeia Commission (IPC), Ministry of Health and Family Welfare, Government of India.

### Primary outcome

**Clinical cure:** clinical cure was defined as asymptomatic and afebrile for more than 48 h, along with USG showing no drainable collection in the liver with removal of the pigtail catheter if any.

### Secondary outcome

1. **Treatment failure**: Treatment failure was defined by the presence of any one of the following condition:
  - Persistently symptomatic even after 72 h of empirical antibiotic and percutaneous aspiration or drainage
  - Development of new collection in the liver during the course of empirical antibiotic therapy
  - Development of shock and new onset organ failure (Encephalopathy, ARDS, AKI) during the course of therapy, leading to shifting to IV antibiotics
  - Culture of the aspirated or drained pus showing growth of microorganism not sensitive to Ciprofloxacin in the Ciprofloxacin-group or Cefixime in the Cefixime-group.
  - Asymptomatic participants requiring persistent drainage or aspiration of the abscess even after 4 weeks of empirical antibiotics therapy
2. **Duration of the therapy:** Number of days of empirical antibiotic therapy to achieve clinical cure
3. **Need for prolong antibiotics therapy:** Asymptomatic participants requiring persistent drainage or aspiration of the abscess even after 2 weeks of empirical antibiotics was given another 2 weeks of extended antibiotic therapy.
4. **Duration of hospital stay:** Number of days receiving in-hospital care
5. **Recurrence of liver abscess:** Development of new liver abscess after clinical cure during the 8 weeks follow up
6. **All-cause mortality:** Any incident of death during the 8 weeks follow up
7. **Incidence of complication:** Development of complications in the form of rupture into peritoneum, pleura, pericardium, development of shock and incidence of new-onset organ failure (Encephalopathy, ARDS, AKI) and need for mechanical ventilation
8. **Need for surgical intervention:** Participants requiring surgical intervention to achieve cure
9. Compliance to the therapy
10. Adverse drug events (ADE)

### Sample size and statistical analysis

**Figure 1:**
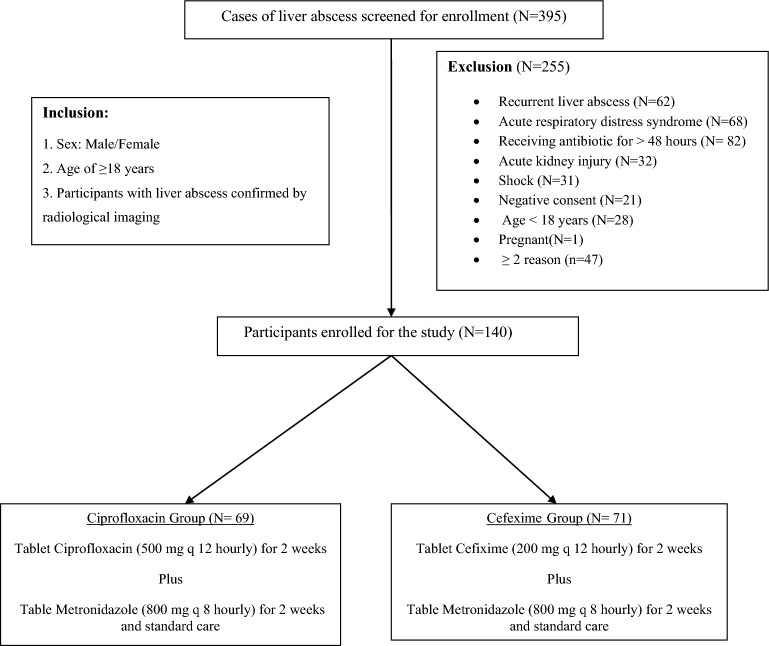
Study design, screening and enrollment.

There was no clinical trial available to predict cure rate or outcome of liver abscess with different regimen of empirical antibiotics therapy. The mortality from liver abscess varied from 10 to 20% in different series^1–3^. As per institutional data base, around 200 patients of liver abscess was expected to be admitted at EMOPD per year. Anticipating at least 60% response rate with a difference of 20% between the groups the total sample size was 128 (64 each group), when there was 80% (power) chance of detecting a significant difference between the two groups at a two-sided 0.05 significance level (α-error of 5%). Anticipating 10% dropout total 140 patients were recruited for the study. Randomization was done with the help of computer generated random number in a block randomization pattern in a block of 10 patients (5:5). Randomization, allocation and treatment concealment was done by the pharmacologist. Both the participants and the principal investigator were kept unaware of treatment allocation. Total of 395 cases of liver abscess were screened for enrollment in the study, out of which 255 cases were excluded as per exclusion criteria (Fig. 1). Eventually total 140 participants were enrolled in the study and randomly assigned into Ciprofloxacin (N = 69) and Cefixime (N-71) groups to receive therapeutic intervention as per study protocol (Fig. 1). The data was managed in data base system through Microsoft Excel and the statistical analysis was performed by SPSS 21.0 version. The analysis was based on available cases. The parametric data was analyzed by paired or unpaired “t”-test, however, the categorical endpoints was analyzed with non-parametric chi-squire test. The p-value of less than 0.05 was considered statistically significant.

## Result

### Baseline characteristics of the study population

The mean age (SD) of the study population was 39.5 (± 13) years with mean body mass index (BMI) of 22.2 (± 2.2) kg/m^2^ and majority of them were male (87.9%) than female (12.1%). Out of 140 participants 7 (5.0%) had diabetes, 3 (2.1%) had hypertension and 112 (80.0%) of them had history of alcohol abuse as per CAGE questionnaires. The distribution of age, sex, BMI and co-morbidity were similar between the groups (Table 1). Out of 140 patients 35 (25%) patients already received antibiotic for 0.4(± 0.8) days before enrollment in the study (Table 1), which was comparable between the groups (p = 0.568).

**Table 1:** Baseline characteristics of the study population.

Parameters	Ciprofloxacin (N = 69)	Cefixime (N = 71)	P value
Age (Mean ± SD years)	39.3 ± 13	36.8 ± 12.3	0.692
Male sex (N = 123)	63 (91%)	60 (84%)	0.218
Female sex (N = 17)	6 (9.8%)	11 (15.4%)	0.218
BMI (Mean ± SD kg/m^2^)	22.4 ± 2.2	22.0 ± 2.1	0.318
Hypertension (N = 3)	1 (1.5%)	2 (2.8%)	1.000
Diabetes (N = 7)	4 (5.8%)	3 (4.2%)	0.717
Alcohol abuse (N = 112)	51 (73.9%)	61 (87.9%)	0.760
Received antibiotic (N = 35)	17 (24.6%)	18 (25.3%)	0.922
Duration of antibiotic (Mean ± SD days)	0.3(± 0.6)	0.5(± 0.9)	0.568
Pain abdomen (N = 135)	68 (98.6%)	67 (94.4%)	0.192
Fever (N = 119)	56 (81.1%)	63 (88.7%)	0.210
Hepatomegaly (N = 79)	41 (59.4%)	38 (53.5%)	0.482

### Clinical features

Most common clinical features were abdominal pain or tenderness (96.4%) followed by fever (85%) and hepatomegaly in 56.4% participants (Table 1). Only 4 (2.8%) participants had clinically recognizable jaundice or yellowish discoloration eyes. Other uncommon presentation was nausea with vomiting in 5 participants and shortness of breath, cough and chest pain in 3 participants each.

### Investigation

Mean (± SD) haemoglobin (Hb), total leukocyte count (TLC) and platelet count of the study population were 11.46(2.2) g/dL, 15.4(5.7) × 10^9^/L, 212.5(140.9) × 10^9^/L respectively. Mean (± SD) serum Bilirubin, Alanine transaminase (ALT) and Aspartate transaminase (AST) were 1.5(1.7) mg/dL, 42.9(36.2) U/L and 44.0(52.7) U/L respectively. In coagulation profile, mean (± SD) Prothrombin Time (PT), Partial Thromboplastin Time (APTT), Prothrombin Time Index (PTI) and international normalized ratio (INR) were 10.0(3.2) seconds, 29.4(3.9) seconds, 73.2(15.5) % and 1.3(0.2) respectively. Most common abnormal finding in blood investigation were anaemia (Hb < 13 g/dL for male or < 12 g/dL for female) in 78.6% (N = 110) participants, followed by coagulopathy in 68.5% (N = 96), leukocytosis (TLC > 11 × 10^9^/L) in 53.5% (N = 75) and hyperbilirubinemia (serum bilirubin > 1.2 mg/dL) in 22.1% (N = 31) and transaminitis (OT/PT > 45 U/L) in 21.4% (N = 30) participants, which were comparable between the groups (Table 3). Beside these 2.1% (N = 3) participants had leucopenia (TLC < 4 × 10^9^/L) and thrombocytopenia (platelet count < 150 × 10^9^/L) and thrombocytosis (platelet count > 450 × 10^9^/L) were found in 5.0% (N = 7) participants each (Table 2).

**Table 2:** Investigation finding of the study population.

Parameters	Ciprofloxacin (N = 69)	Cefixime (N = 71)	P value
Anaemia (N = 110)	54 (78.2%)	56 (78.8%)	0.930
Leucocytosis (N = 75)	35 (50.72%)	40 (56.3%)	0.506
Leucopenia (N = 3)	2 (2.8%)	1 (1.4%)	0.543
Thrombocytosis (N = 7)	4 (5.7%)	3 (4.0%)	0.669
Thrombocytopenia (N = 7)	3 (4.3%)	4 (5.6%)	0.727
Hyperbilirubinemia (N = 31)	12 (17.3%)	19 (23.7%)	0.182
Transaminitis (N = 30)	16 (31.4%)	14 (21.2%)	0.617
Coagulopathy (N = 96)	47 (68.1%)	49 (69.0%)	0.909
Pus culture positive (N = 9)	4 (5.8%)	5 (7.0%)	0.763
Pus culture grew *E. coli* (N = 4)	1 (1.4%)	3 (4.2%)	0.324
Ciprofloxacin resistance (N = 6)	2 (2.9%)	4 (5.6%)	0.424
Cefixime resistance (N = 2)	1 (1.4%)	1 (1.4%)	0.983
Amoebic Trophozoite (N = 1)	0	1 (1.4%)	0.507
Amoebic Serology positive (N = 64)	34 (49.2%)	30 (42.2%)	0.404

Out of 140 participants, 133(95.0%) participants underwent pigtail Catheter drainage (PCD)/single time aspiration (STA) of the liver abscess. Only 9 (6.4%) participant’s pus-culture showed growth of organisms. Most common organism grew was *E. coli* in 4 samples, out of which only one was sensitive to Ciprofloxacin and three were resistant to Ciprofloxacin. One participant each had growth of *Proteus mirabillis* (resistant Ciprofloxacin), *Klebsiella pneumoniae* (resistant to Ciprofloxacin), *Enterobacter cloacae* (sensitive to Ciprofloxacin), *Staphylococcus aureus* (sensitive to linezolid/teicoplanin/clindamycin/vancomycin) and pseudomonas Aeruginosa (sensitive to ciprofloxacin). Overall out of nine cases, 6 had resistance to ciprofloxacin (2 in Ciprofloxacin-group and 4 in Cefixime-group). Cefixime resistance organism grew in 2 cases, one for *E. coli* and one for *Klebsiella pneumoniae*, which were also resistant to Ciprofloxacin. Amoebic serology was positive in 64 (45.7%) participants, though amoebic trophozoite could be found under microscopy in one sample only. Microorganism and Amoebic serology both were positive for 4 (2.8%) participants.

### Characteristic of the liver abscess

Mean number (± SD) of liver abscess in the study population was 1.2(0.4) which was similar between the groups (p = 0.238). Majority of the participants (86.4%, N = 121) had single liver abscess and 12.1% (N = 17) participants had double abscesses where as only 1.4% (N = 2) participants had multiple (> 2) liver abscesses. Most common location for the liver abscess was right lobe (72.8%), followed by left lobe (26.4%) and the caudate lobe was involved in one participant only. As per size of the abscess majority of the participants (87.8%) had liver abscess of > 5 cm in size, 10.7% participants had liver abscess of 3–5 cm in size and only 1.4% participants had liver abscess of < 3 cm in size. The characteristics of the liver abscess were comparable between the groups (Table 3).

**Table 3:** Characteristic of the liver abscess and intervention.

Parameters	Ciprofloxacin (N = 69)	Cefixime (N = 71)	P value
Number of abscess	1.10 ± 0.349	1.24 ± 0.92	0.238
Single abscess (N = 121)	63 (91.3%)	58 (81.7%)	0.096
Double abscess (N = 17)	5 (7.2%)	12 (16.9%)	0.080
Multiple abscess (N = 2)	1 (1.4%)	1 (1.4%)	0.983
Caudate lobe abscess (N = 1)	1 (1.44%)	0	0.493
Left Lobe abscess (N = 37)	22 (31.8%)	15 (21.1%)	0.149
Right Lobe abscess (N = 102)	46 (66.6%)	56 (78.8%)	0.103
Size of abscess < 3 Cm (N = 2)	2 (2.8%)	0	0.241
Size of abscess 3–5 Cm (N = 15)	9 (13.0%)	6 (8.4%)	0.380
Size of abscess > 5 Cm (N = 123)	58 (84.0%)	65 (91.5%)	0.195
STA (N = 6)	4 (5.8%)	2 (2.8%)	0.372
PCD (N = 122)	59 (85.5%)	63 (88.7)	0.376
Both PCD & STA (N = 5)	1 (1.4%)	4 (5.6%)	0.192

STA, Single time aspiration; PCD, Pigtail catheter drainage (PCD).

### Intervention

Along with standard medical therapy, majority (95.0%) of the participants (N = 133) required USG guided intervention either pigtail catheter drainage (PCD) or single time aspiration (STA). Out of 140 participants, most common intervention was PCD alone (87.1%, N = 122) followed by STA (4.3%, N = 6) and 5(3.6%) participants required both STA & PCD of the abscesses. The USG guided interventions were comparable between the groups (Table 3).

## Outcome

### Primary outcome

Out of 140 participants, majority (89.3%) of them (N = 125) achieved clinical cure. Though clinical cure was more in Cefixime group (93.0%, N = 66) as compared to the Ciprofloxacin group (85.5%, N = 59), the difference was not statistically significant (p = 0.154).

### Secondary outcome

Out of 140 participants, 15 (10.7%) participants could not achieve clinical cure. The most common reasons for the treatment failure was persistent hepatic collection requiring drainage of pus through PCD for more than 4 weeks leading to prolong antibiotic therapy in 13 (9.3%) participants followed by new-onset shock and new-abscess formation in one (0.7%) participant each (Table 4). Among this participants (N = 15), aspirated/drained pus from two participants in Ciprofloxacin group grew Ciprofloxacin-resistant microorganism and one participants in Cefixime group grew microorganism not sensitive to Cefixime also attributing to treatment failure (p = 0.542). Persistent drainage of pus through PCD for more than 4 weeks leading to treatment failure was significantly higher (p = 0.036) in Ciprofloxacin group (14.5%, N = 10) as compared to the Cefixime (4.2%, N = 3) group (Table 4).

**Table 4:** Outcome of the empirical oral antimicrobial therapy.

Outcome parameters	Ciprofloxacin (N = 69)	Cefixime (N = 71)	P value
Clinical Cure (N = 125)	59 (85.5%)	66 (93.0%)	0.154
2 weeks therapy (N = 82)	40 (58.0%)	42 (59.1%)	0.886
Extended therapy (> 2 weeks, N = 43)	19 (27.5%)	24 (33.8%)	0.421
Duration of therapy (mean ± SD, days)	15.1 ± 4.5	16.0 ± 4.2	0.223
Duration of hospital stay (mean ± SD, days)	1.6 ± 0.7	1.5 ± 0.6	0.210
Treatment Failure (N = 15)	10 (14.5%)	5 (7.0%)	0.154
Persistent drainage for > 4 Weeks (N = 13)	10 (14.5%)	3 (4.2%)	0.036
New-onset shock (N = 1)	0	1 (1.4%)	0.322
New-abscess Formation (N = 1)	0	1 (1.4%)	0.322
Accidental slippage of pigtail catheter (N = 3)	3 (4.3%)	0	0.075
Readmission (N = 5)	3 (4.3%)	2 (2.8%)	0.625
Poor compliance (N = 5)	4 (5.8%)	1 (1.4%)	0.161
Adverse drug reaction (N = 92)	44 (63.8%)	48 (67.6%)	0.632
Nausea (N = 49)	26 (37.7%)	23 (32.4%)	0.512
Gastritis (N = 29)	13 (18.8%)	16 (22.5%)	0.589
Numbness (N = 14)	10 (14.5%)	4 (5.6%)	0.080

Mean (± SD) duration of empirical oral antimicrobial therapy to achieve clinical cure was 16.2 (4.3) days which was similar (p = 0.223) between the groups (Table 4). Total 82 (58.6%) participants required only 2 weeks of empirical oral antimicrobial therapy to achieve clinical cure, 40 (58.0%) in Ciprofloxacin group and 42 (59.1%) in Cefixime group, which was also comparable (p = 0.886) between the groups (Table 4). Whereas, total 43 (30.7%) participants required extended duration (> 2 weeks) of empirical oral antimicrobial therapy to achieve clinical cure, 19 (27.5%) in Ciprofloxacin group and 24 (33.8%) in Cefixime group, which was also comparable (p = 0.421) between the groups (Table 4).

Mean (± SD) duration of hospital stay was 1.6(0.6) days, which was similar (p = 0.210) between the groups (Table 4). The participants were followed up for 8 weeks and none of them had recurrence of liver abscess and there was no mortality either. Total 5 (3.6%) participants required readmission. One participant had new-abscess formation and three participants had accidental slippage of pigtail catheter requiring PCD drainage. One participants was readmitted because of new-onset shock and was managed successfully with two days of in hospital intravenous hydration, antibiotic and supportive care. There was no incident of rupture of liver abscess and none of the participants required surgical intervention.

Over all compliance to the therapy was very good. Compliance to the therapy was inadequate in only 5 (3.6%) participants, who stopped taking medication because of the side effect 4 (5.8%) in Ciprofloxacin group and 1 (1.4%) in Cefixime group (Table 4). They were managed symptomatically and completed the therapy and follow-up after further counseling.

The drugs were well tolerated and no serious adverse events were observed during the 8 weeks of follow up. Overall, 92 (65.7%) participants reported mild ADR, which was similar (p = 0.632) in the Ciprofloxacin (63.8%, N = 44) and Cefixime (67.6%, N = 48) groups (Table 4). Most common ADR was nausea in 49(35.0%) participants, followed by gastritis in 29(20.7%) and numbness of fingers in 14(10.0%), which were similar between the groups (Table 4). Nausea and gastritis related symptoms could be managed successfully with antacid or proton pump inhibitor. The numbness was self-limiting, resolved spontaneously after stopping medication and required no specific additional therapy.

## Discussion

Liver abscess is a medical emergency which can be potentially catastrophic if not promptly intervened with antimicrobials and percutaneous drainage/aspiration. With the modern advancement in microbiology, imaging and intervention techniques, the mortality from liver abscess has significantly decreased in past decades ranging from 10 to 30% and reported as low as 2.5% of cases^6^.

Based upon microbial-etiology liver abscess is categorized as either amoebic or pyogenic. In developing countries like India amoebic liver abscess is still more common than pyogenic liver abscess all though there is an ascending trend of pyogenic liver abscess in recent decade^7,8^. Previously, *Escherichia coli* was the most common organism causing pyogenic liver abscess, which is replaced by *Klebsiella pneumoniae* worldwide, accounting for 50–70% of cases in the Asian subcontinent^9,10^. Other common organisms causing pyogenic liver abscess are Streptococcus species, Enterococcus, Anaerobes such as Bacteroides and Peptostreptococcus, and other gram-negative organisms^9,10^. Liver abscess due to Poly-microbial (≥ 2 microorganism) etiology was also not uncommon and reported up to 24% cases^6,7^.

The mainstay of treatment is prompt antimicrobial with or without percutaneous drainage/ aspiration of the abscess. Whether antibacterial or amoebicidal is to be given is determined based on amoebic serology & pus culture reports, which are mostly unavailable initially at the time of presentation. Many cases of liver abscess are due to poly-microbial infection and microbial etiology is indeterminate in many cases also^6,7^. Hence empirical antimicrobials with both antibacterial and amoebicidal agents along with percutaneous drainage/ aspiration is the standard care for liver abscess^11^. Though percutaneous drainage procedures have significantly improved the prognosis of liver abscess and curtailed the need for surgical intervention, there is no clear consensus or studies regarding the standard protocol for empirical antimicrobials regarding choice, route of administration or duration of therapy. Presently, there is no randomized control clinical trial available to evaluate or compare the efficacy of empirical antimicrobial regimens for the treatment of liver abscess. Antibiotics recommendations are based upon the common microbial etiology and local bacterial resistance patterns when available. The empirical antimicrobial regimen should cover enteric gram-negative bacilli, anaerobes and streptococci. The empirical antimicrobial regimen should also cover *E. histolytica* until microbial etiology is found or amoebic abscess is excluded. The recommended empirical broad-spectrum parenteral antimicrobial regimens are a third or later generation cephalosporin, a Beta-lactam-beta-lactamase inhibitor combination, a Carbapenem with or without Metronidazole^12,13^. The commonly preferred antibiotics are parenteral Ceftriaxone, Piperacillin-tazobactam, Imipenem and Meropenem. There is not much evidence supporting the rational for upfront use of intravenous (IV) antibiotics for the management of uncomplicated liver abscess. There is also lack of clinical trial regarding the efficacy of oral antimicrobials for the treatment of uncomplicated liver abscess. The present RCT tried to evaluate and compare the efficacy of empirical oral antimicrobial regimens (Ciprofloxacin plus Metronidazole and Cefixime plus Metronidazole) for the treatment of uncomplicated liver abscess.

### Clinical spectrum

In the present study male and female sex ratio was more than 7:1, suggesting males were at higher risk for liver abscess compared to females and mean age of the participants was 39.5 ± 13.0 years, indicating liver abscess predominantly occurred in third or fourth decade which were similar to the previously conducted institutional study^3^. The most common clinical presentation was abdominal pain/tenderness (96.4%) followed by fever (85.0%) and hepatomegaly (56.4%). Jaundice (2.8%) was less commonly reported as a presenting complaint but hyperbilirubinemia was found in 22.1% (N = 31) participants. This clinical presentation was similar to previous studies^3,14^. Similar to the previous study, present study also demonstrated that majority of liver abscesses were solitary (86.4%) and mostly involved right lobe (72.8%) of the liver^15^. However, two or more abscess were present in only 19(13.6%) participants as compared to previous studies reporting multiple liver abscess in 20–25% cases^15^. This could be due to the fact that, the present study included only uncomplicated liver abscesses, since complications are more likely with multiple liver abscesses. However the characteristics of liver abscesses were similar between the Ciprofloxacin & Cefixime group.

Based on microbial etiology, 45.7% participants had amoebic liver abscess, 6.4% had pyogenic liver abscess, 2.8% had mixed infection and etiology was indeterminate in 47.8% cases. The incidence of pyogenic liver abscess was lower as compared to the previous study reporting 21% cases^7^. The lower number of pyogenic abscess could be because of 25% of participants had already received antibiotics for 0.421(± 0.81) days before enrollment in the study and all participants underwent percutaneous procedure at around 6 h after hospitalization during which they received empirical antibiotics, possibly leading to lower growth of microorganism in the pus culture. In the present study only 9(6.4%) participants had a growth in pus culture and most common organism was *E. coli* (44.4%). Previous Indian studies also reported *E. coli* being the most common organism causing pyogenic liver abscess, followed by *Klebsiella pneumoniae*, whereas in other Asian countries most common organism was *Klebsiella pneumoniae*^7–10^. While considering amoebic serology for the diagnose amoebic liver abscess it should be noted that false negative results can occur occasionally in the early course of the disease, due to delay in rise of antibody titer and false positive results can occur in the background of subclinical amoebic infections which can be avoided by considering high titer for diagnosis^16^.

### Outcome

Out of 140 participants almost 90% (N = 125) participants achieved clinical cure with empirical oral antimicrobial therapy (Table 4). Though the clinical cure rate was higher in Cefixime group (93.0%, N = 66) as compared to Ciprofloxacin group (85.5%, N = 59), the difference was insignificant (p = 0.154). As per the present study both Cefixime plus Metronidazole and Ciprofloxacin plus Metronidazole were very efficacious empirical oral antimicrobial regimen for the treatment of uncomplicated liver abscess and this also prevented nonessential prolong in-hospital care and intravenous medications. Till date there is no clinical trial available to evaluate and compare the efficacy of empirical oral Cefixime or Ciprofloxacin with Metronidazole for the treatment of uncomplicated liver abscess. Recently Molton et al.^17^ realized that oral antibiotics were non-inferior to IV antibiotic for the treatment of liver abscess caused by *Klebsiella pneumoniae*. But the study was open label, included only *Klebsiella pneumoniae* related pyogenic liver abscess, majority of the participants received IV antibiotics for median 5 days and more than 50% of participants underwent drainage of abscess prior to the enrollment. In the present study, patients who already received antibiotics for more than 48 h at the time of presentation were excluded. In the present study, only 25% of participants received antibiotic for 0.4 (± 0.8) days prior to the enrollment in study and underwent abscess drainage/aspiration prospectively after enrollment as per indication. Nearly 10% (N = 15) participants had treatment failure, 10 (14.5%) in Ciprofloxacin group and 5(7.0%) in Cefixime group, which was comparable (p = 0.154) between the groups (Table 4). Most common reason for treatment failure was persistent hepatic collection requiring drainage and prolong antibiotic therapy for more than 4 weeks in 13(9.3%) participants, which was significantly (p = 0.036) higher in Ciprofloxacin (14.5%, N = 10) group compared to the Cefixime (4.2%, N = 3) group (Table 4). As per the present study Oral Cefixime appeared to be better than oral Ciprofloxacin in term of achieving more clinical cure and lesser chance of clinical failure due to persistent collection requiring drainage for more than 4 weeks leading to prolong antibiotic therapy, but this would need to be investigated further in a larger clinical trial.

There is insufficiency of data regarding the optimum duration of antibiotic therapy for the treatment of liver abscess. Most studies used a regimen of 2 weeks of parenteral, followed by more prolong course (4–6 weeks) of antimicrobial therapy and switching to oral antibiotics when clinical and inflammatory responses allowed^18^. According to the present study mean (± SD) duration of antimicrobial therapy to accomplish clinical cure was 16.2 ± 4.3 days which was lower contrast to the previously published studies^17–19^, where the duration of antibiotic therapy was 4–6 weeks. This could be due to, study included only pyogenic liver abscess and lesser number of patients underwent drainage procedure which could explain the longer course of antibiotics. In the present study, the mean duration of antimicrobial therapy was 15.1 ± 4.5 days in Ciprofloxacin group & 16.0 ± 4.2 days in Cefixime group (p = 0.223). According to the present study 2–3 weeks of oral antimicrobial therapy with or without percutaneous drainage/aspiration was adequate for the treatment of uncomplicated liver abscess. Further studies comparing the efficacy of empirical oral and intravenous antimicrobial for the treatment of liver abscess are encouraged. Duration and choice of antimicrobial and the route of administration should be individualized as per response to therapy and presence or absence of complications.

Giangiuli et al.^20^, in a retrospective study of pyogenic liver abscess observed that, 30-day readmission rate was significantly higher in patients who received transition to oral (IV followed by oral) antibiotics (39.6% Vs 17.6%) than continued IV antibiotics. Whereas in present study overall only 5 participants required readmission, 3 participants had accidental slippage of PCD, 1 participant had new onset shock and 1 had new abscess formation. The same study also uncovered that 60-days recurrence rate of liver abscess was similar both in transition to oral antibiotic (8.3%) and continued IV antibiotics (9.8%) groups^20^. In the present study none of the participants experienced recurrence of abscess during the 8 weeks of follow up. This indicated oral antibiotic therapy did not increase the risk of recurrence as compared to IV antibiotic therapy.

During the follow up no participants required surgical intervention due to complications like rupture into pericardium/ peritoneum/pleural cavity and there was no mortality. However in retrospective analysis morality from pyogenic liver abscess was reported around 10% and amoebic liver abscess was 2–15%^3,19^. The need for surgical intervention due to complication was reported around 7% in pyogenic liver abscess and 2–7% in amoebic liver abscess^3,19^. This could be explained by the fact that the present study included only uncomplicated liver abscess cases and majority of the participants (95%) underwent prompt percutaneous aspiration/drainage.

Oral ciprofloxacin has a bioavailability of 70% and the calculated absolute bioavailability of Cefixime was 48%, however following oral administration mean peak plasma concentrations of cefixime was 2.0–2.6 mg/l after a single dose of 200 mg/tablet, whereas for ciprofloxacin mean peak serum concentrations after single doses of 500 mg/tablet was 1.51 to 2.91 µg/ml, which were similar and usually attained in 3–4 h^21,22^. In previous studies, tissue penetration of ciprofloxacin was excellent and certainly it was a unique feature of fluoroquinolones compared to other antibiotics. It was also observed that body fluid and tissue concentrations of Ciprofloxacin equaled or exceeded those in concurrent serum samples; exceptions being bronchial secretions, cerebrospinal fluid and saliva^21^. In case of Cefixime it was shown to have a concentration of 99 mg/l at 4.5 h after single dose in common bile duct in patients with biliary tract infection^22,23^. However, the concentration of both the drugs in pus of liver abscess is not available yet and further research is encouraged in this aspect.

It is also important to note that resistance to ciprofloxacin is on the rising trend due to inappropriate and irrational use of antibiotics. As per Ali et al.^24^ clinical isolate of 27% *E. coli*, 22% *Staphylococcus aureus*, 72% *Klebsiella pneumoniae*, 16% *Salmonella typhi* and 44% *Pseudomonas aeruginosa* were resistant to ciprofloxacin. These are the common causative organism for pyogenic liver abscess or secondary infection associated with amoebic liver abscess. A previous studies with cefixime stated that in comparisons with other antibacterial drugs the in vitro potency of Cefixime against Enterobacteriaceae was greater than that of Cefaclor and Cephalexin whereas it was less active than Ciprofloxacin against all tested species of Enterobacteriaceae^22,23^. In a clinical trail Cefixime was found superior to ciprofloxacin for the treatment of community acquired pneumonia^25^. Whereas in our study we observed that clinical cure was higher in Cefixime group (93.0%) compared to the Ciprofloxacin group (85.5%) but statistically insignificant (p = 0.154) and other secondary end points were also similar between the groups (Table 4). But in Cefixime group there was significantly (p = 0.036) lesser chance of treatment failure due to persistent collection requiring drainage for more than 4 weeks leading to prolong antibiotic therapy.

### ADR and compliance

Over all the compliance was very good as it was oral therapy. As the majority of the previously published studies were retrospective in nature, adherence to therapy could not be evaluated or compared. Out of 140 patients, 5 participants had inadequate compliance, however they were re-counseled and they completed the therapy and follow up.

Most common ADR was nausea in 49 (35.0%), followed by gastritis in 29 (20.7%) and numbness of fingers in 14 (10.0%) participants, which were similar between the groups. Commonly reported ADR with Ciprofloxacin were related to the gastrointestinal tract (7.8%), nausea, vomiting and diarrhoea and central nervous system (3.3%), dizziness, tremors and headache^21^. Commonly reported ADR with Cefixime were diarrhea (13.5%) and stool changes^22^. Commonly associated ADR with Metronidazole were dry mouth (47%), abdominal pain (21%), nausea (9%)^26^. All the three drugs used in our study Ciprofloxacin, Cefixime and Metronidazole could have caused nausea as reported in previous studies. Most common ADR associated with all the three drugs were also gastrointestinal symptoms. Numbness was reported in 14 participants, probably due to Metronidazole as seen in previous studies, due to mechanism like free radical damage, axonal degeneration of nerve fibers by inhibiting protein synthesis or nutrition deficiencies^27^. ADR reported in the study were self limiting and were reversible with symptomatic treatment and stoppage of the drug.

Limitations of the study were, it was a single center clinical trial, raising doubt about the representation of large diverse Indian population. However, PGIMER being a tertiary care center caters to multiple states which include Punjab, Haryana, Himachal Pradesh, Uttarakhand, Uttar Pradesh, Jammu & Kashmir, Rajasthan and Bihar, representing large and diverse parts of India. Sample size of the study was small. Microbiological etiology of the liver abscess could not be determined in large proportion (47.8) of the participants as all the participants received empirical antimicrobials therapy for some duration before they underwent percutaneous drainage/ aspiration procedure possibly leading to lower growth of microorganism in the pus culture. As per expectation the primary endpoint /cure rate was higher with Cefixime, as there is growing antimicrobial resistance to ciprofloxacin, but it did not reach statistical significance. The sample size may have been inadequate to prove small but clinically meaningful improvement of cure rate with Cefixime than Ciprofloxacin. Further multicentre studies with large sample size are encouraged.

Intravenous administration of drug is superior to oral administration in term of achieving greater drug level and prompt action. But at the same time intravenous administration needs expertise and greater resources preferably in-hospital care with extra cost of drugs and additional financial burden to the patients as well as healthcare system. In resource constraint setting at the emergency, receiving number of patients beyond capacity, many patients even cannot afford to purchase expensive intravenous medications, items required for the percutaneous intervention and the additional cost of in-hospital care. Oral medicines with adequate bioavailability are blessing for the people of the poor and developing countries. Both ciprofloxacin and Cefixime have good oral bioavailability and recently Molton et al.^17^ also found that oral antibiotics were non-inferior to IV antibiotic for the treatment of liver abscess. So, the selection of drugs, route of administration and time frame of the antimicrobial therapy ideally should be customized and adapted as per presence or absence of complications and response to the therapy.

In conclusion, both, the Ciprofloxacin or Cefixime plus Metronidazole for duration of 2–3 weeks were efficacious as empirical oral antimicrobial regimen along with prompt percutaneous drainage or aspiration for the treatment of uncomplicated liver abscess. Both, Ciprofloxacin and Cefixime had similar efficacy for achieving clinical cure. Oral Cefixime was better than oral Ciprofloxacin in term of achieving lesser chance of treatment failure due to persistent collection, leading to prolong antibiotic therapy which is required to be investigated further in larger clinical trial. Choice, route of administration and duration of the antimicrobial therapy for the treatment of liver abscess should be individualized as per presence or absence of complications and response to the therapy.

## Data Availability

All reasonable data requests should be submitted to the corresponding author (DPD) for consideration.
